# How do patients, medical assistants and physicians accept and experience tablet-based cognitive testing by medical assistants in general practice? - A qualitative study

**DOI:** 10.1186/s12875-025-02823-z

**Published:** 2025-05-17

**Authors:** Kristin Rolke, Carolin Rosendahl, Klaus Weckbecker, Alexander Hanke, Michael Wagner, Leon Nissen, Lara Marie Reimer, Stephan Jonas, Philipp Schaper, Jochen René Thyrian, Florian Schweizer, Judith Tillmann

**Affiliations:** 1 Institute of General Practice and Primary Care, Chair of General Practice I and Interprofessional Care, Witten/Herdecke University, Alfred-Herrhausen-Str. 50, 58448 Witten, Germany; 2 Department of Old Age Psychiatry and Cognitive Disorders, University Hospital Bonn, Venusberg- Campus 1, 53127 Bonn, Germany; 3 Institute for Digital Medicine, University Hospital Bonn, Venusberg-Campus 1, 53127 Bonn, Germany; 4 Department of Psychology, Psychological ageing research, University of Siegen, Adolf-Reichwein-Str. 2a, 57068 Siegen, Germany; 5 German Center for Neurodegenerative Diseases (DZNE), site Rostock/Greifswald, Ellernholzstr. 1-2, 17489 Greifswald, Germany; 6 Institute for Community Medicine, University Medicine Greifswald, Ellernholzstr. 1-2, 17489 Greifswald, Germany; 7 Faculty V, University of Siegen, Adolf-Reichwein-Str. 2a, 57068 Siegen, Germany; 8 TUM School of Computation, Information and Technology, Technical University of Munich, Arcisstraße 21, 80333 München, Germany

**Keywords:** Dementia, GP care, Timely diagnosis, Tablet-based testing, Digitalisation, Family medicine, Medical assistant, Cognitive disorders

## Abstract

**Background:**

Approximately 1.8 million people with dementia live in Germany and the number is expected to increase in the coming years. Between 360,000 and 440,000 new cases are diagnosed each year. General practitioners (GPs) are often the first point of contact for people with concerns about their memory performance or already noticed symptoms of dementia. However, structural barriers can hinder timely diagnosis by GPs, resulting in diagnoses frequently being made later in the disease’s progression. Tablet-based cognitive testing, carried out by medical assistants (MAs) in GP practices, is being tested in the iCreate feasibility study, and could facilitate detection of dementia, allowing those affected to receive timely treatment and support. However, the acceptance, user experience and perceived benefits and consequences of routine implementation of such a not established procedure remain unclear until now.

**Methods:**

In this qualitative study, seven GPs, six MAs and eight patients were qualitatively interviewed regarding the acceptance, user experience of the tablet-based procedure and its implications for GP care. Semi-structured interviews were conducted using newly developed guidelines, recorded, transcribed and analysed according to Kuckartz and Rädiker using MAXQDA.

**Results:**

All respondent groups had a positive perception of the digital testing in GP practices. Interviewed MAs welcomed the new responsibilities, and patients gladly accepted the opportunity of cognitive assessment in response to their memory concerns. GPs supported delegating additional tasks to MAs. Patients found the digital testing tasks feasible to complete on the tablet and MAs also had positive experiences using the tablet as test administrators. All groups can generally envision a long-term implementation of the tests in practice, but also noted possible barriers, like the need for additional communication with specialists, limited time resources, and currently insufficient remuneration of cognitive testing.

**Conclusions:**

The positive user experience and high acceptance of participants indicate that tablet-based cognitive testing in GP settings can be highly feasible and can thus lead to indicated specialist referrals. Consequently, the management of patients exhibiting dementia symptoms should increasingly commence in GP practices, receive adequate funding, and occur in close collaboration with other specialized disciplines.

**Supplementary Information:**

The online version contains supplementary material available at 10.1186/s12875-025-02823-z.

## Background

Dementia, a leading cause of disability and dependency among older adults worldwide, presents a significant public health challenge. According to the World Health Organization (WHO), more than 55 million people globally were living with dementia in 2020, with nearly 10 million new cases diagnosed each year [1]. An increase up to 132 million by 2050 is predicted by the WHO [2]. In Germany, the prevalence of dementia is equally concerning, with approximately 1.8 million individuals affected [3]. Dementia is an umbrella term for a range of diseases and injuries that impact the brain, most of which are progressive in nature [2]. Alzheimer’s disease is the most common form of dementia, accounting for 60–70% of cases [2].

Timely diagnosis of dementia is essential: It allows for intervention and information, which can help manage symptoms, slow disease progression, and improve the quality of life for patients and their families through support and care, even if there is no cure. The best time to make a diagnosis is when effective treatment or support concepts can be applied (timely diagnosis) [4, 5]. Despite its importance, timely diagnosis remains challenging due to the subtle and often overlooked initial symptoms of dementia [6]. Research suggests that as many as half of people living with dementia have never been diagnosed [7].

In the German healthcare system, general practitioners (GPs) play a pivotal role, often serving as the primary point of contact for patients [8]. They are responsible for a wide range of healthcare services and are frequently the first professionals to encounter patients with dementia symptoms, making them crucial in the initial diagnostic process. However, people with dementia are frequently undiagnosed in primary care [9, 10].

The German Federal Ministry of Health highlights the crucial role of GPs in the first diagnosis and assessment of dementia [11]. The S3-guideline on dementia recommends that GPs administer cognitive short tests such as the MMSE (Mini-Mental-State-Examination) or the MoCA (Montreal Cognitive Assessment) when there are indications of dementia reported by the patient or at the GP’s active request [5]. Generally, GPs should collaborate with specialists in neurology, psychiatry and psychotherapy to conduct comprehensive neurological and psychiatric diagnostics [11]. Radiologists may also be involved in the diagnostic process [12]. However, regulations regarding the responsibilities in dementia management vary across Europe [13]. In Germany, ensuring adequate care for people with dementia poses a significant challenge to the healthcare system, resulting in considerable complexity for both patients and their caregivers [14]. There is an urgent need for research to address these challenges of timely dementia identification by making diagnostic testing more accessible [15]. Despite these recommendations, dementia testing and assessment in general practices in Germany is often not conducted consistently enough [16], so dementia is often not or belatedly recognised [17]. The reasons for this include time constraints and high workloads in the practices which make time-consuming tests challenging. Additionally, initially difficult-to-recognize symptoms [18], a lack of resources and training leading to widespread direct referrals to specialists [19], patients and their relatives concealing signs out of shame [20] or fear and stigmatisation [21, 22] regarding dementia as well as inadequate monetary compensation play a role [19, 23, 24]. In addition to the already mentioned aspects of the generally high workload in German GP practices, which is further exacerbated by a continuous shortage of doctors [25], the division of labor within the practice teams could contribute significantly to alleviating the burden. In this context, the role of medical assistants (MAs, German: Medizinische Fachangestellte (MFA)) in general practices is becoming increasingly significant. Traditionally, MAs have been involved in administrative tasks, patient management, and basic clinical duties such as taking vital signs and preparing patients for examinations [26]. However, this project aims to expand their role in Germany in conducting dementia screening tests using tablets. This approach seeks to enhance the efficiency and accuracy of preliminary dementia assessment in GP practices and could support GP practices in the timely identification and management of patients with cognitive impairments. The involvement of non-physician professionals in the diagnosis of dementia has been increasingly focussed on for some time in the literature [27, 28]. To date, there are only a limited number of studies on dementia care provided by GPs in Germany, particularly concerning the potential role of MAs in the timely diagnosis of dementia. Furthermore, the available studies are predominantly qualitative studies [29, 30]. They indicate a high level of self-motivation among MAs to support the recognition of dementia in their practices [29]. Additionally, trained MAs have demonstrated the ability to improve patient care, including in the context of dementia [28]. Despite the insights presented and the interest of the MAs in taking on additional responsibilities within their practice, such instances are, however, rare in Germany. The extent to which MAs can be concretely integrated into the diagnostic process and how this could happen in daily practice will be explored based on the study.

Digital cognitive testing is increasingly being utilized and can achieve comparable diagnostic results to traditional pen-and-paper methods [31]. Digital versions offer greater accessibility and cost efficiency [32], immediate automated data scoring and interpretation and the possibility of using the test in a self-administered manner [33], leading to time savings among healthcare professionals [34]. The MoCA is a 10-minute screening tool for cognitive impairment (mild cognitive impairment (MCI) and dementia) [35]. Several studies highlight that the MoCA is particularly suitable for recognising early and mild stages of dementia [5], better than the commonly used MMSE [35, 36]. A validated digital MoCA version for tablets is commercially available [37]. The use of a digitally realised version of the MoCA in combination with delegation of tasks to MAs could optimise the diagnostic process.

The present paper focuses on the qualitative evaluation results of the iCreate study (“Digitally supported case finding to improve the diagnosis and care of patients with dementia in primary care”), a study that aims to assess the feasability, validity and clinical impact of tablet-based cognitive testing by MAs in GP practices. The qualitative evaluation specifically highlights acceptance, user experience, and perceived benefits and consequences of routine implementation in GP practices from the perspectives of patients, MAs, and GPs. The aim is to demonstrate the extent to which the three interviewed groups accept the tablet-based testing and how manageable it is from the user’s perspectives. Additionally, the study covers their views about implementation into the daily routines of GP practices.

## Methods

### Contextualisation of the study in the iCreate-project

This qualitative analysis is part of the evaluation of the iCreate study, a prospective feasibility study introducing a tablet-based, digital cognitive screening procedure in general practitioner’s offices. iCreate is conducted from June 1, 2023, to May 31, 2025, by a consortium of four university institutes and one research institution in North Rhine-Westphalia, western Germany.

The iCreate study evaluates several aspects of implementing a digital version of the Montreal Cognitive Assessment (MoCA), administerd by trained MAs subsequent to a request by the GP, into practice routine in *n* = 10 general practices in Bonn and the Rhine-Sieg district. General practices were recruited through personal outreach and received detailed information on the study. The MAs received a two-hour on-site training by a trained professional on how to administer the tablet-based MoCA, further study aspects, general information on dementia and working with people with dementia. In addition, practical test runs were conducted with several test scenarios to practice using the assessments in real situations. Throughout the project, there was regular contact between the practices and the project team, with MAs having a point of contact for both technical and professional questions. The patients were recruited through the study materials (posters and flyers), as well as through personal contact.

Each participating GP practice received a tablet with a pen, a licensed version of the MoCA Duo App [38] and a newly developed iCreate-App containing the quantitative evaluation (for usability) for MAs and patients as well as a software solution to securely transfer the data. The tests were carried out on site at the practices and lasted approx. 25 min (including pre- and post-test discussions, e.g. filling out the consent forms, and the quantitative evaluation part). In order to compensate the practices for additional tasks and resulting time consumption, they received an expense allowance per tested patient (15€ for the MAs and 25€ for the practice).

The app automatically summarized test results and allows the GP to potentially suggest further action based on the outcomes: additional examinations at the memory clinic of the University Clinic in Bonn, follow-up testing in twelve months or no further procedures. To facilitate further examination at the memory clinic, study patients were given priority through reserved time slots.

The Ethics Committee of the Medical Faculty of the University of Bonn, Germany, (No. 258/23-EP), granted approval for the whole iCreate feasibility study. The Ethics Committee of the Witten/Herdecke University granted approval for the qualitative study presented in this article (No. 266/2023).

### Study design

This paper focuses on the qualitative evaluation component of the iCreate-study. The further methodological information is based on the COREQ-guidelines [39].

Qualitative individual interviews were conducted between February and July 2024. Theoretical saturation (additional interviews are not generating any more information) has been selected as the criterion to stop the recruitment process for interviewees. The recruitment and conduct of the interviews were carried out by two researchers who were in close communication regarding all content and jointly determined the point of theoretical saturation which has been reached after the following number of qualitative interviews within each group: GPs (*n* = 7), patients (*n* = 8) and MAs (*n* = 6). These interviews were conducted in 7 out of 10 practices. Two patients had to cancel their planned interview appointment due to health restrictions. One MA was also unable to attend an interview appointment due to the high staff shortage in the practice. New interviewees were then recruited for the cancelled interviews.

The patients as interview participants were recruited via the participating GP practices either by the practices staff or they were directly called by the project staff after receiving written consent. The interviewed GPs and MAs worked in the mentioned practices and were recruited via phone by the project staff. The interviews were conducted by two female interviewers (with M.Sc. Public Health and M.Sc. Therapy Sciences) in German- both by telephone and face-to-face in the GP practices. The telephone interviews were a valued alternative for the interview partners since GPs and their teams were usually short of time. Since participants could choose the interview setting (face-to-face vs. telephone), it is assumed they were in a comfortable atmosphere, which positively impacted the interview and encouraged an open flow of narration. The preparation and follow-up also helped build trust, ensuring no noticeable differences in data quality. However, as with all telephone interviews, aspects like facial expressions and non-verbal behavior could not be assessed. The interviewers were trained and experienced professionals in designing, conducting and analyzing qualitative interviews with patients and experts in the healthcare sector with experience of working with groups with complex needs, including older people and refugees. Patients were included in the interviews if they met the following inclusion criteria:

- 60 years or older
- Patient in one of the ten participating GP practices
- Participated in the iCreate cognitive testing
- fluent in German

Recruitment efforts have been made to have a fairly balanced number of men and women as well as diversity of cognitive impairment levels among interviewed patients. Participants received a written information sheet about the study with information on data protection (e.g. regarding audio recording of the interview) as well as information on cognitive testing, signed an informed consent form and filled in a short questionnaire about sociodemographic information. Additionally, all participants were orally informed by the interviewers about the qualitative study details. The interviews with patients were conducted after they had completed the cognitive test and the subsequent consultation with their GP.

### The interview guide

Based on literature and in exchange with the interdisciplinary project team three semi-structured interview guides (patient, MA, GP) were developed, based on the guidelines by Helfferich [40]. The newly developed interview guides were evaluated by the whole, interdisciplinary iCreate project team which includes neuropsychologists, health services researchers, psychologists, general practitioners, therapy scientists, computer scientists and epidemiologists. Each interview guide was pretested once and no changes were needed. The three interview guides can be seen in Appendix 2. A summary of main topics of the three interview guides can be seen in Appendix 1 and are equivalent to the main categories. Patients with dementia or suspected cognitive impairment and older people in general constitute a vulnerable interview group. This has been taken into account during development of the patient interview guide. Two blocks of questions about dementia (e.g.: “The test you took allows us to detect dementia and dementia-related changes at an early stage. How did you feel about it?”) were not asked unless the patient raised the issue.

The average interview duration was as follows: GPs (18 min); MAs (15 min); Patients (17 min). All interviews were audio-recorded and fully transcribed by an external service provider. Quality control of transcripts was done afterwards by the project team. Further details about the interview atmosphere or incidents during the interview were noted in interview protocols.

### Data analysis

The transcribed interviews were analysed using the qualitative content analysis method according to Kuckartz and Rädiker [41] with the computer software MAXQDA, version 24. The interview material was analysed with the content-structured approach. Codes were developed both deductively (subject areas from interview guides) and inductively (directly from interview material) as seen in Appendix 1. Coding of the interviews was done by two authors independently who discussed and compared the results afterwards to ensure good quality and objectivity. Based on the previously created codes, a category system was developed that structured the codes hierarchically into main and subcategories (Appendix 1). A systematic analysis of the material was then carried out based on this system.

### Sample characteristics

All MAs interviewed were female, five were aged between 18 and 34 years and one between 45 and 59 years. Four female and three male GPs participated for an interview, mainly aged 35–59 years (6 out of 7). The patients recruited were balanced in gender (4 female and 4 male) and all aged over 60 years (60–69 years: *n* = 2, 70–79 years: *n* = 5, 80–89 years: *n* = 1).

The interviewed patients regularly use technical devices both privately and professionally. Laptops/ computer and smartphones were used by all particpants. Half of the patients interviewed were familiar with using a tablet. This also applied for the majority of MAs interviewed (66%).

## Results

The results of the interviews with GPs, MAs and patients are based on three main topics: “Acceptance” means to what extent the interviewees appreciate and accept the tests, e.g. how the expansion of tasks for MAs is assessed, what motivation patients had to participate and how teamwork has changed for GPs through the delegation of tasks. “User experience” describes how testing with the tablet was practicable for MAs and patients. “Perceived benefits and consequences of routine implementation” contains the extent to which respondents could see this type of testing being carried out in GP practices in long term, and what requirements should be met or would be desirable.

### Acceptance

#### Medical assistants

Some of the MAs interviewed stated that the tablet testing was a new task that they enjoyed and which adds variety to their everyday work. The additional patient contact was also perceived positively.

*“Personally*,* I enjoy doing these tests. Simply because it’s one more area of responsibility that adds a bit of variety to my working life. But I also think it’s nice to have another opportunity to work with patients.” (MA 1)*

The MAs reported receiving positive feedback from patients about their role as test administrators. This could be seen by the MAs when they were directly praised for their empathy in the test situation or when patients sought dialogue and shared their concerns. Patients and MAs were often familiar with each other through regular visits to the GP practice so that patients confided in the MAs.

*“I’ve actually received consistently positive feedback so far. (…) that I’ve done well.” (MA 4)*

The MAs also reported that patient contact in this situation also required empathy and time, which may be lacking in the doctor-patient contact.

*“Because I think*,* especially when we MAs do this*,* the connection between patient and MA is different to that with the doctor. Because we are perhaps a bit more*,* I don’t want to say more empathetic*,* but we communicate with the patient on a different level than the doctor. Because for the patient*,* the doctor is always the one who makes the diagnosis*,* who*,* intervenes a bit more in the patient’s life. And that may not be the same case with the MAs.” (MA 1)*

#### Patients

The patients interviewed stated that they had decided to undergo the test voluntarily and had become aware of it through notices in their GP practices. Their reasons for participating included concerns about a perceived decline in cognitive performance, fear of potential consequences and examples of dementia within their family and/or among acquaintances. The majority of patients interviewed would recommend the test to others, as it offers the opportunity to address dementia-related diseases at an early stage.

*“Because I have noticed that I forget things about myself very quickly. As I mentioned*,* sometimes I can’t remember*,* from one room to the next*,* what I was actually doing here. That scares me a bit.” (P 4)*

The patients perceived the tests carried out by an MA as pleasant and competent. The delegation of such tasks to a MA was welcomed, particularly because some patients had been visiting the GP practice for a long time and were already familiar with the MA. This familiarity often led to a relationship of trust, making patients feel well supported during the test.

*“I think that’s completely normal. Why does it always have to be a doctor? It doesn’t have to be. (…)They (MAs) probably spend more time with the patient and interact more closely than the doctor does*, *who is always under time pressure (…).” (P 5)*

#### General practicioners

The GPs interviewed reported receiving positive feedback regarding the testing from both the patients and the MAs. For patients who had noticed subjective declines themselves (e.g. forgetfulness), the test was seen as helpful in assessing the state of their memory. The GPs then had the opportunity to offer further care steps as part of the project.

“*My experiences have been very positive. Patients respond very enthusiastically. Some see the poster that’s displayed outside. But most come to us*,* let’s say*,* inductively with their concerns*,* and that’s very good when we can immediately offer a procedure. Patients are also very grateful that a direct flow is created right away and that there is a clear programme. Because they come with a lot of uncertainty. It gives them the security that everything (the testing) is structured. Based on my experience so far*,* this works very well.” (GP 3)*

In general, the doctors supported the delegation of the MoCA testing to their MAs. Tablet testing was described as a structured process with predefined procedures and questions, which helped free up time of medical staff. In addition, the interviewees described that working with the MAs fostered a different level of teamwork, as MAs were involved in the diagnostic process and their work was valued in a new way. This interaction allowed MAs to gain a better understanding of the clinical picture of diseases like dementia and to better understand the work and workload of the doctors.

*“Well (…)*,* delegating tasks ultimately leads to good teamwork. I always find it quite gratifying that the MAs feel they’ve accomplished something and can help us in the diagnostic process*,* which definitely enhances their role. Of course*,* it frees up our time. The patients appreciate it. They somehow have the feeling that it’s a standardised procedure (…)*,* someone sits there alone with them in the room. And it’s separate from the conversation with the doctor. I think it has a lot of advantages in our process. It is good.” (GP 2)*

### User experience

#### Medical assistants

Two of the MAs interviewed had previously carried out memory tests on paper. However, the majority had no prior experience with memory tests in their GP practice. In general, the MAs reported positively on the ease of use and implementation of the tablet-based test. The more often the MAs administered the test, the more confident they became in using it:

*“At first*,* of course*,* you’re still a bit nervous because you think: ‘Oh no*,* I hope I don’t make a mistake’*,* whether it’s forgetting a step or needing to review the final result or possibly making a mistake. But I think you become more confident from patient to patient.” (MA 2)*

Some MAs were suprised to find out that older aged patients were also able to handle the tablet well and did not encouter problems. Only a few patients inquired the option of solving the task using pencil and paper.

*“(…) and generally to see how the patients react to it*,* because they are often older patients*,* for whom tablets are sometimes still completely foreign. I thought that (tablet-testing) was really great.” (MA 2)*

One of the tasks was identified by several MAs as being particularly error-prone. This task involved using the tablet’s microphone to recognise spoken words. The tablet’s microphone didn`t work perfectly in recognizing spoken words.

*“Well*,* actually everything is self-explanatory. I explain to them once at the beginning that they can erase it in certain situations or rewrite it*,* and otherwise it’s self-explanatory. The only issue is that when patients need to say words with an F*,* the system doesn’t save them. This means you have to enter it manually later (…).” (MA 4)*

In addition, technical difficulties arosed due to an unstable internet connection. According to one MA interviewed, some task instructions were accasionally not immediately understandable for patients, so she paraphrased or rephrased them in her own words.

### Patients

The interviewed patients found the tablet to be pleasant and easy to use, rating its handling positively. Some patients had practised using a tablet beforehand, but even those unfamiliar with this technology managed well.

*“ I found this tablet very pleasant. I didn’t know how to use it before. And it’s very easy to use and quite good (…).” (P 1)*

For the interviewed patients the tasks on the tablet were generally suitable. Some found the tasks more challenging, e.g. due to limited time for a task, while others found them easier.

*“There are easy questions*,* there are difficult questions. And the only thing was that you had to complete tasks in a certain amount of time. And I have to say*,* if you’re thinking and thinking and then you run out of time*,* you can feel a certain amount of time pressure (…)“ (P 8)*

For some tasks, patients had to draw directly on the tablet using the provided pencil. All interviewees reportet being successfull with this task.

*“I was a bit shaky then. You had to take a square and extend it into three dimensions. And the lines were shaky*,* but the task as itself was all good (…) “(P 7)*

### General practicioners

The doctors could only provide limited information on the user experience of the tablet because their MAs administered the tests. Some described the test as a very successful tool for delegation to MAs, as it already provides a lot of structure. The MAs who conducted the tests were almost exclusively in the 18–34 age group. One interviewed doctor explained delegating the task to younger MAs by being more ‘tech-savvy’ than their older colleagues. Older patients were also estimated to be quite confident in handling and using the tablet for testing.

*“I think it’s basically a very good tool for delegation*,* because the tablet already specifies a lot of things. In other words*,* you provide a semi-structured tool. As I said*,* I think the tablet should actually be able to do this on its own. Because the usability*,* as Google and Apple have already shown*,* even very elderly people can be very well guided by such a system.” (GP 2)*

Tablet testing was preferred over paper-based testing in the interviews because, according to one interviewee, it allowed for better planning of the duration and provides a more standardized testing. Such testing could theoretically also be applied to other illnesses.

*“(…) And somehow you have the feeling that it‘s more standardised with the tablet*,* because a lot is predefined and it’s less dependent on the examiner. At least*,* I hope so. Yes*,* so I definitely see advantages. If I had to choose*,* I would favour the tablet-based version because I think the advantages outweigh the disadvantages. It seems more objective. It’s easy to estimate how long it will take.” (GP 2)*

### Perceived benefits and consequences of routine implementation

#### Medical assistants

All MAs could imagine the test being offered and administered in their GP practice in the long term. One MA pointed out the particular relevance of testing with regard to the ageing society:

*“Simply because there’s a relatively large number of people in favour of it. Sure*,* it’s something new now*,* maybe that’s why the demand is so high. (…) But the diseases (…) or the memory disorders themselves are always present. I think that this tablet-based test may well remain a long-term feature of our practice. Simply because the diseases are there. And with the aging population*,* it will continue to play an increasingly important role.” (MA 1)*

Although the testing required staffs time resources, the digital approach with the tablet was welcomed by interviewees. Some MAs mentioned that they see long-term benefits in integrating the test into routine patient care at their GP practices. It allowed the practice to provide more services to patients and enabled quicker referrals to other specialists. It showed patients that GP practices can serve as a point of contact for dementia symptoms.

*“I think it already has a big impact. On the one hand*,* because the testing itself is an additional service we can offer to patients as a practice. But I also think that it adds a bit more to the quality in our practice. Simply because it allows for earlier diagnosis of diseases such as dementia or Alzheimer’s. And we have one more opportunity to connect patients with specialists.” (MA 1)*

**Fig. 1:**
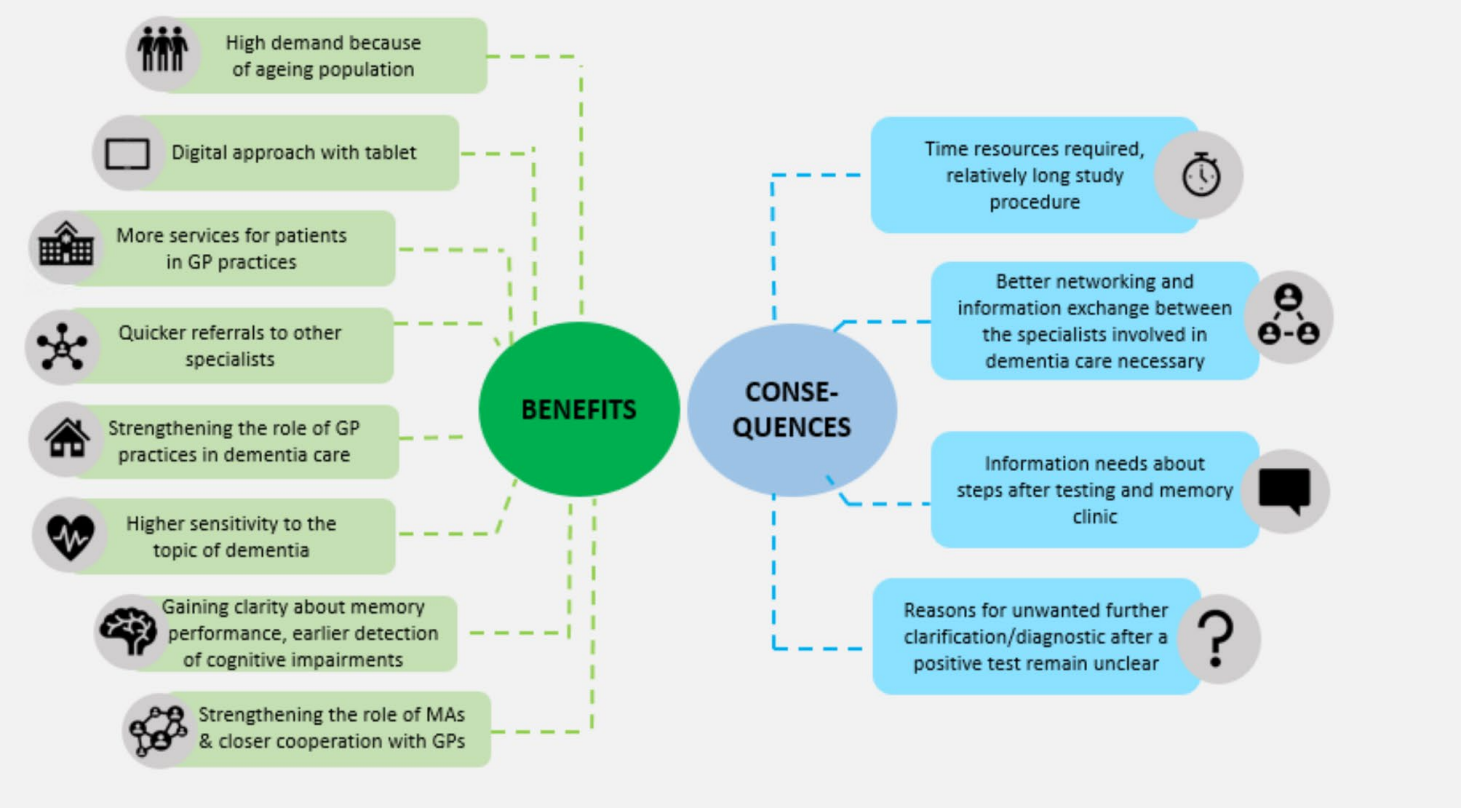
Perceived benefits and consequences concerning long-term implementation of digital memory tests in GP practices, reported by GPs, patients and MAs

One MA also stated that she had benefited from the project, as it made her more sensitive to the topic of dementia in the work context and in interactions with patients. There were also requests for improvements. In addition to the content aspects mentioned earlier, one MA expressed the desire for better networking and information exchange between the various specialists involved in dementia care. GP practices were often not informed about the further steps taken in care of their patients who scored low on the MoCA test. Perceived benefits and consequences for long-term implementation the digital testing in GP practices are shown in Fig. 1.

#### Patients

The majority of interviewed patients would recommend the test to other people in the future. The testing was also compared to a prevention programme to ensure health in future times:

*“(…) Because I’m someone who wants to know what’s going on*,* you know? That’s exactly why I go for cancer screenings or to the gynaecologist or anything else. I want to know what’s going on with me. And I would advise everyone to do the same. The earlier I go*,* the earlier you can do something if there’s an issue. And in my opinion*,* early detection is the most important thing of all.” (P 4)*

The opportunity to gain clarity about one’s own memory performance was seen as positive as it allowed for the possibility of exploring treatment options at an early stage. The test, along with receiving a good result, had a reassuring effect on the respondents.

*“If you’re already concerned about whether you might be among those affected*,* it’s important to take the test so you can put your mind at ease.” (P 3)*

On the other hand, some interviewees expressed a need for more information about the process and the care pathway after testing has been completed. For example, not everyone felt fully informed about what should happen after a test with an abnormal result and what the referral to the memory clinic entails:

*“That someone simply explains what the memory clinic is*,* what they do there*,* and what you need to do. I would just be interested in that.” (P 4)*

#### General practitioners

The interviewed GPs perceived the test as a low-threshold and early offer for patients, providing an opportunity to offer further steps, if necessary. The referral to other specialists like psychiatrists or neurologists to conduct a cognitive short-test was not necessary, which could be a relief for patients.

*“Because the GP offer is already lower-threshold compared to getting a referral to a psychiatrist. People can sometimes be a bit put off by that (referral to psychiatrist)*,* they don’t like that. Which I understand.” (GP 7)*

Interviewees explained that the testing option could also detect cognitive impairments at an early stage and reassure patients and their relatives if the results are unremarkable. Both outcomes are beneficial for patients’ personal planning for the future.

*“Well*,* we can offer low-threshold testing at a very early stage and then*,* if the test results are concerning*,* refer them to a specialist centre very quickly. From my point of view*,* this is very important*,* especially because there is often fear*,* which is often unfounded*,* and patients perceive normal age-related forgetfulness as threatening. They worry that it could progress quickly and actually be the onset of Alzheimer’s dementia. But we can often reassure them and simply explain that it is harmless age-related forgetfulness. And these tests are very helpful for catching and calming patients early.” (GP 2)*

Some doctors were very surprised that certain patients did not want further clarification of their test result. However, the exact reasons were unclear. The interviewees assumed that a poor result in the test situation was too devastating for those affected.

*“I think one person wanted to be referred and most of them then say: ‘Oh no*,* that’s enough for me.’ They don’t want to pursue it further.” (GP 5)*

Case identification also led to a new exchange or a different type of cooperation between MAs and doctors, which was welcomed for the future:

*“In our setting here*,* I think it’s a great collaboration*,* a great opportunity for collaboration*,* not just this*,* one prepares something or does a bit of the hands-on work*,* but this is actually also an exchange that is encouraged through the patients*,* and I always think that’s good.” (GP 2)*

GPs generally found digital solutions and tests beneficial for the future. However, several potential improvements were identified: Some GPs perceived the current test as too long, leading to patients fatigue, and ties up staff resources that are not practical for everyday work in the long term and are not adequately compensated. Ideally, a shorter test with high sensitivity and specificity that can be performed by the patient independently, was considered the optimum, mentioned by two GPs. In addition, a digital and direct transfer of the results to patient management systems would be useful. This would allow the results to be viewed and available without the need to access the tablet.

*“We need digital solutions to ultimately be able to care for more patients with the same amount of manpower. Because that’s what we’re facing (…) We can only achieve this through multipliers*,* i.e. abolishing useless bureaucracy and utilising multiplication instruments. And this is where tablet-based testing can really help*,* but not if a person has to stand next to it. Then we can only do one test. That’s not where I see the benefit of the tablet.” (GP 1)*

## Discussion

The acceptance of tablet-based dementia testing in GP practices, as identified in this qualitative evaluation, can be considered high. All stakeholders involved in the procedure reported a positive impression: the MAs welcomed the testing as a valuable addition to their tasks, the GPs supported delegating the testing to the MAs, and patients appreciated the accessible nature of the service. In the qualitative results, the user experience of the tablet test was generally rated positively, although a few areas for improvement were identified. The staff administering the test was able to effectively instruct the patients and operate the tablet. The tasks were also considered manageable for the patients interviewed. As outlined, the doctors had limited interaction with the tablet and relied on feedback from both patients and their MAs. Both MAs and GPs considered the long-term use of the tablet testing to be feasible. However, this will necessitate further improvements, particularly in the collaboration among different specialist disciplines and by shortening the time for the tests.

### Acceptance

The delegation of tablet-based testing to MAs was well-received by the stakeholders involved. The MAs themselves appreciated the opportunity to contribute to dementia diagnosis and expand their expertise. As indicated in other studies, MAs expressed a general interest in participating in dementia diagnostics [16]. However, this is still rarely implemented, largely due to the absence of standardized procedures [42]. In this study, testing was facilitated by a tablet that provided a structured framework with standardized tasks and questions. Training for MAs, combined with the frequent use of this tool, also helped to reduce uncertainties among the participating MAs. More efforts should be made to train MAs and involve them in diagnostic processes such as dementia diagnosis in line with their interests and skills.

GPs could save time and simplify their work by delegating testing to their staff; however, they note that this still consumes resources from MAs. Given the shortage of doctors in Germany, particularly in the GP sector [25], it is increasingly important to alleviate the pressure on currently practicing GPs. In recent years, the role of Physician Assistants (PAs) has been increasingly discussed in the literature. Although training opportunities for PAs have existed for a long time and job satisfaction within this professional group is very high, PAs remain underrepresented in Germany to date [43, 44]. Further possibilities to reduce the commitment of personnel resources in GP practices should be discussed and tested.

Further studies also confirm that patients are generally open to expand medical tasks to MAs [45]. While acceptance of testing at their GP practice appears to be high in our study, it remains unclear why some patients with positive test results refuse to pursue further diagnostics. The doctors interviewed could only speculate about the reasons, such as being unable to make an appointment themselves or feeling discouraged or disappointed by the initial results. Another possible explanation is that patients may require information about treatment options because they may think that nothing can be done about this incurable disease (see results) [46]. The fear of receiving a dementia diagnosis, along with concerns about the associated stigma [20], may contribute to a reluctance to seek or accept a diagnosis. Further research into these reasons is urgently needed, as the current situation leads to canceled care, uninvestigated causes other than Alzheimer’s, and a complete breakdown of the care pathway. On the other hand, it is ultimately up to the patients to decide whether they wish to pursue an initial suspicion or, given their current situation, prefer not to know the status of their health (right not to know) [47, 48].

### User experience

All interviewees described the user-friendliness of the tablet test as good, providing important insights into its future feasibility within standard care. The minor technical issues mentioned regarding tablet use should be checked and remedied in the future. Additionally, the structured testing process with the tablet proved beneficial for the MAs, leading to increased confidence with each test conducted through repetitive procedures. The MAs conducting the tests, as well as all interviewed patients, had prior experience using technical devices, including tablets. However, not everyone in these age groups in Germany (over 60 years old) possesses such knowledge. A survey conducted in 2020 revealed that only 20% of (*n* = 1.075) respondents over the age of 65 reported using a tablet (42% laptops and 41% smartphones) [49]. Technical devices are frequently used for internet access in Germany, but there are notable differences among older individuals in Germany based on education level and access. On average, those with a higher level of education use the internet much more frequently than those with lower educational attainment. Additionally, an individual’s professional background and prior relationship with technical devices also influence their usage patterns [50]. Moreover, a greater affinity for technology was noted among younger MAs, likely due to their upbringing with these devices or earlier exposure. However, strategies should be developed for widespread implementation to help all MAs in practices to foster skills and interest in new technologies.

### Perceived benefits and consequences of routine implementation

The practice staff interviewed acknowledged the benefits of the memory testing service for routine patient care. Both patients and staff described the testing application as easily accessible. Additionally, the practice staff became more aware of dementia-related illnesses as a result of their involvement in the program.

In Germany, dementia is primarily diagnosed by specialists. Many GPs refer patients to the appropriate specialist disciplines when dementia is suspected [30, 51]. However, this additional referral can pose a challenge for patients exhibiting dementia symptoms, as highlighted in the interviews, often requiring support from relatives or friends. Improved (digital) networking between GPs and specialists, as considered in the project, along with feedback on treatment steps provided to the referring GP, is crucial for ensuring comprehensive care. Strengthening the role of GP practices through standardized tools such as the MoCA via tablet can help to establish them as low-threshold key points of contact for dementia-related diseases and symptoms. The use of digital apps in general practice can play a significant role in future diagnostics, as recent studies have shown [52].

Enhanced training and continuing education opportunities for general practice personnel can improve the care situation for individuals with dementia, as early signs of memory impairment can be more effectively recognized [16]. GPs with specialized geriatric training in dementia care demonstrate greater knowledge in this area, which facilitates more accurate diagnoses [53]. This expertise can be further enhanced through networking with local and community-based care services [54]. Dementia care managers can play a crucial role in linking these elements, acting as intermediaries between doctors, patients, and their families. Recent pilot projects have confirmed that such initiatives lead to greater stability in care for physicians and provide a sense of security when dealing with diagnostics [55].

The tests conducted as part of the project resulted in the identification of early symptoms of dementia, or even advanced stages, in some patients who had previously been unaware of their condition. This recognition allowed for the initiation of further steps in their care. In the long term, this creates opportunities for patients and their families to plan ahead, enabling them to maintain independence for as long as possible [56, 57]. Additionally, it provides the possibility of managing symptoms with medication and slowing the progression of the disease [56].

The test was perceived as lengthy by the GPs and MAs interviewed. Note that, as part of the feasibility study, the actual MoCA testing was followed by several questions regarding its usability (quantitative results from a larger sample of patients will be reported elsewhere). These additional questions would not be asked in a standard care situation. On average, the MoCA proper test took 13 min to complete.

Time is described as a significant constraint in nearly all practices, which is why GPs are open to digitized tests which can be applied by MAs. Automated analyses of these tests, along with the transmission of results, offer advantages when utilizing digital tools, even if the interpretation of results is left to specialized staff. Options for more time saving solutions should be investigated in more detail in future studies.

### Limitations

One of the limitations of this study is the selection of interview participants among the patients. Only individuals who expressed interest participated in the interviews and preliminary tests, whereas those who found the experience unsettling opted not to participate. Moreover, the interviewees were very open to the topic and had sought further diagnostics. In cases of unremarkable findings, they highlighted the positive aspect of gaining certainty about their memory performance and potentially preventing an illness at an early stage, while scarcely addressing themes of shame or stigmatization in connection with cognitive testing. This may be attributed to the fact that questions regarding dementia were only explored in depth when the interviewees themselves introduced the topic.

Furthermore, it can be assumed that the practices participating in the study were already sensitized to the topic of dementia, which may have influenced how these topics were discussed with the patients. Additionally, the materials (flyers and posters) used to approach potentially interested patients for memory testing in general practices were in German. This likely excluded individuals with limited German proficiency, as well as those with visual or reading impairments, unless they received direct, personal outreach from the practice team. The interviews with the participants were relatively short. On the one hand, a limited amount of time was assumed, especially by GPs and MAs, when creating the interview guide. On the other hand, in practice, participants’ responses on some topics were shorter than expected, such as regarding the previously mentioned topic of dementia.

The recruitment and inclusion of practices for this project were concentrated in Bonn and the Rhein-Sieg district, primarily due to their proximity to the collaborating memory clinic at University Hospital Bonn. Some patients opted not to pursue further diagnostics after scoring low on the MoCA tests in their GP practices. The reasons for this decision were only partially documented. Additionally, all interviewed patients were comfortable using technical devices, which may not be representative of the broader age group in Germany.

## Conclusions

The study offers valuable insights into the acceptance of digital tablet testing by MAs as support for timely dementia diagnostics in general practices. The findings demonstrate a positive user experience and acceptance, and highlight the perceived benefits and consequences of routine implementation of digital tools in GP settings. Given the rising number of individuals with dementia in Germany, timely and accessible testing is particularly important. Therefore, the care of patients exhibiting dementia symptoms should start in the general practice environment. Utilizing digital testing with tablets administered by MAs represents a promising approach for timely identification of patients, ultimately enhancing overall care. This increased awareness of dementia among both the practice team and patients is crucial.

While the current study has shown high acceptance of tablet based testing, the patient-related benefits (e.g. diagnoses after specialist consultation, initiation of treatment) and the improvement of networking among specialists require further research.

## Electronic supplementary material

Below is the link to the electronic supplementary material.

Supplementary Material 1

Supplementary Material 2

## Data Availability

The datasets generated and analysed during the study are available from the corresponding author on reasonable request.

## Abbreviations

GP: General practitioner
NRW: North-Rhine-Westphalia
MA: Medical assistant
MoCA: Montreal Cognitive Assessment
MMSE: Mini Mental State Examination
PA: Physician assistant
