# Correction: How do patients, medical assistants and physicians accept and experience tablet-based cognitive testing by medical assistants in general practice? - A qualitative study

**DOI:** 10.1186/s12875-026-03271-z

**Published:** 2026-04-14

**Authors:** Kristin Rolke, Carolin Rosendahl, Klaus Weckbecker, Alexander Hanke, Michael Wagner, Leon Nissen, Lara Marie Reimer, Stephan Jonas, Philipp Schaper, Jochen René Thyrian, Florian Schweizer, Judith Tillmann

**Affiliations:** 1 Institute of General Practice and Primary Care, Chair of General Practice I and Interprofessional Care, Witten/Herdecke University, Alfred- Herrhausen-Str. 50, Witten, 58448 Germany; 2 Department of Old Age Psychiatry and Cognitive Disorders, University Hospital Bonn, Venusberg- Campus 1, Bonn, 53127 Germany; 3 Institute for Digital Medicine, University Hospital Bonn, Venusberg- Campus 1, Bonn, 53127 Germany; 4 Department of Psychology, Psychological ageing research, University of Siegen, Adolf-Reichwein-Str. 2a, Siegen, 57068 Germany; 5 German Center for Neurodegenerative Diseases (DZNE), site Rostock/ Greifswald, Ellernholzstr. 1-2, 17489 Greifswald, Germany; 6 Institute for Community Medicine, University Medicine Greifswald, Ellernholzstr. 1-2, Greifswald, 17489 Germany; 7 Faculty V, University of Siegen, Adolf-Reichwein-Str. 2a, Siegen, 57068 Germany; 8 TUM School of Computation, Information and Technology, Technical University of Munich, Arcisstraße 21, München, 80333 Germany

**Correction: BMC Prim. Care 26**,** 174 (2025)**

10.1186/s12875-025-02823-z

Following publication of the original article [1], an incorrect statement was inserted in the "Contextualisation of the Study in the iCreate Project" section during the proofing process.

The updated statement is given below and the changes have been highlighted in **bold typeface.**

***Incorrect***:

Each participating GP practice received a tablet with a pen, a licensed version of the MoCA Duo App [38] and a newly developed iCreate-App containing the quantitative evaluation (for usability) for MAs and patients as well as a software solution to securely transfer the data. The tests were carried out on site at the practices and lasted approx. 25 min (including pre- and post-test discussions, e.g. filling out the consent forms, and the quantitative evaluation part). In order to compensate the practices for additional tasks and resulting time consumption, **its the other way round**,** 15€ for the MAs and 25€ for the practice.**

***Correct***:

Each participating GP practice received a tablet with a pen, a licensed version of the MoCA Duo App [38] and a newly developed iCreate-App containing the quantitative evaluation (for usability) for MAs and patients as well as a software solution to securely transfer the data. The tests were carried out on site at the practices and lasted approx. 25 min (including pre- and post-test discussions, e.g. filling out the consent forms, and the quantitative evaluation part). In order to compensate the practices for additional tasks and resulting time consumption, **they received an expense allowance per tested patient (15€ for the MAs and 25€ for the practice).**

The original article [1] has been corrected.
