# Supplementary material for: How do patients, medical assistants and physicians accept and experience tablet-based cognitive testing by medical assistants in general practice? - A qualitative study
Source: BMC Prim Care. 2025 May 17;26:174. doi: 10.1186/s12875-025-02823-z (PMC12085061; doi:10.1186/s12875-025-02823-z)
Supplement: Supplementary file 1 — Supplementary Material 1 [file 12875_2025_2823_MOESM1_ESM.pptx]

## Slide 1
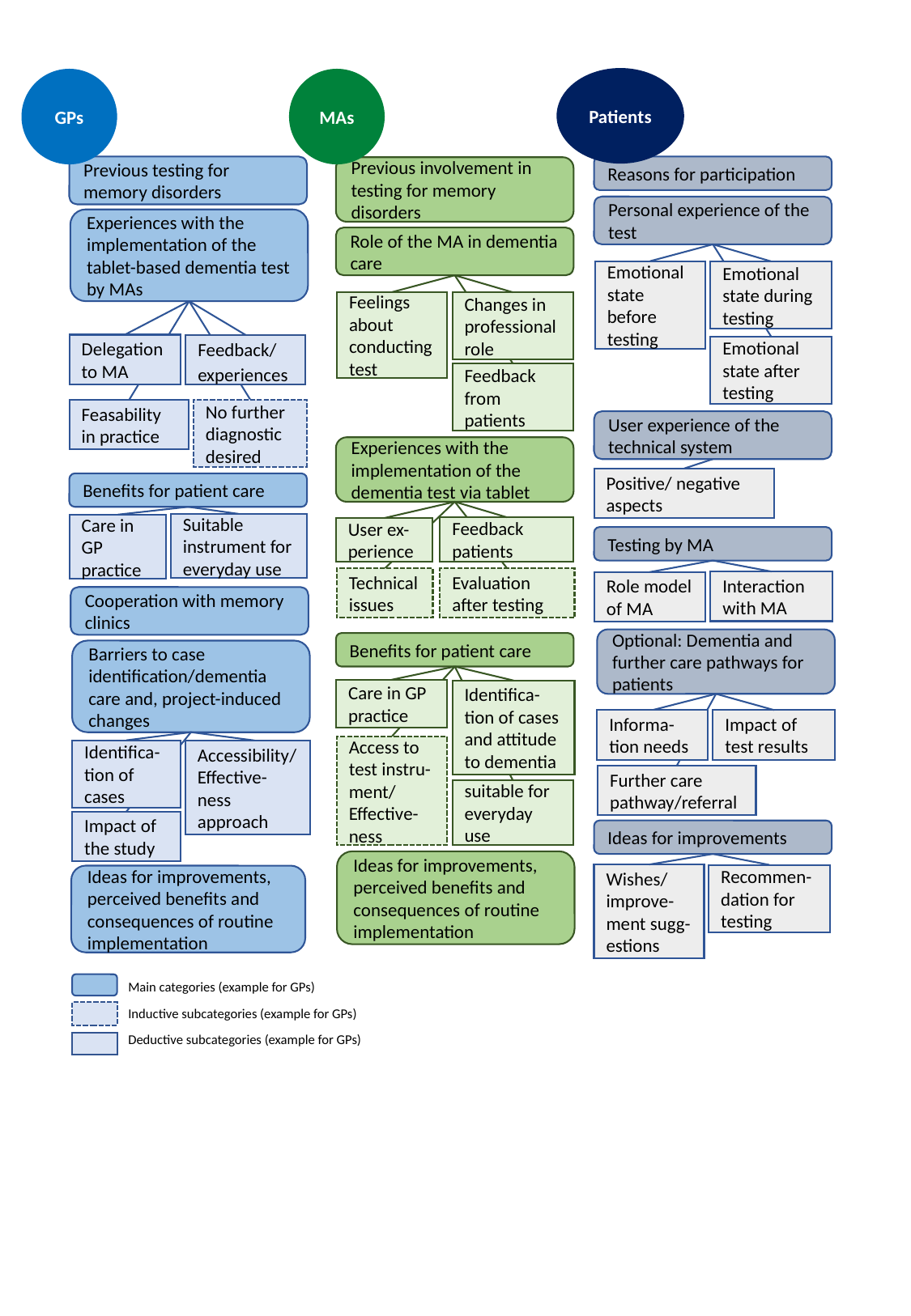

Patients
GPs
MAs
Previous testing for memory disorders
Reasons for participation
Previous involvement in testing for memory disorders
Personal experience of the test
Experiences with the implementation of the tablet-based dementia test by MAs
Role of the MA in dementia care
Emotional state before testing
Emotional state during testing
Feelings about conducting test
Changes in professional role
Delegation to MA
Feedback/ experiences
Emotional state after testing
Feedback from patients
No further diagnostic desired
Feasability in practice
User experience of the technical system
Experiences with the implementation of the dementia test via tablet
Positive/ negative aspects
Benefits for patient care
Suitable instrument for everyday use
Care in GP practice
Feedback patients
User ex-perience
Testing by MA
Technical issues
Evaluation after testing
Interaction with MA
Role model of MA
Cooperation with memory clinics
Optional: Dementia and further care pathways for patients
Benefits for patient care
Barriers to case identification/dementia care and, project-induced changes
Care in GP practice
Identifica-tion of cases and attitude to dementia
Impact of test results
Informa-tion needs
Access to test instru-ment/ Effective-ness
Identifica-tion of cases
Accessibility/ Effective-ness approach
Further care pathway/referral
suitable for everyday use
Impact of the study
Ideas for improvements
Ideas for improvements, perceived benefits and consequences of routine implementation
Wishes/ improve-ment sugg-estions
Recommen-dation for testing
Ideas for improvements, perceived benefits and consequences of routine implementation
Main categories (example for GPs)
Inductive subcategories (example for GPs)
Deductive subcategories (example for GPs)
