# Supplementary material for: How do patients, medical assistants and physicians accept and experience tablet-based cognitive testing by medical assistants in general practice? - A qualitative study
Source: BMC Prim Care. 2025 May 17;26:174. doi: 10.1186/s12875-025-02823-z (PMC12085061; doi:10.1186/s12875-025-02823-z)
Supplement: Supplementary file 2 — Supplementary Material 2 [file 12875_2025_2823_MOESM2_ESM.docx]

**Interview guide: GPs (main questions)**

**Opening question:**

Since the winter of 2023, you and your practice have been participating in our study and have now had the opportunity to implement the testing with patients. Before you share your experiences with us, we would like to know whether and to what extent you have previously conducted cognitive testing in the care of people with memory disorders, such as dementia, in your practice.

**Experiences with the implementation of the dementia test via tablet by the medical assistant (MA):**

- How did you perceive the implementation so far? What worked well, and what woked less well?

**Benefits for primary care and testing by the medical assistant (MA):**

- What impact do you think the study has on patient care in general?
- What benefits can you identify for your practice?
- Do you consider dementia testing by the MA in this context a suitable tool for everyday practice? If yes/no, why/why not?
- How do you assess the possibility of involving MAs in dementia diagnostics to support you? What works well, and what is challenging/difficult? What effect does delegating the testing to the MA have on everyday practice?

**Barriers to case identification/dementia care and, project-induced changes**

- What has specifically changed in case identification and referral due to the study?
- What impact has the study had on identifying potential dementia patients who might have otherwise gone unnoticed?
- How has participation in the study influenced your personal attitude towards the dementia condition and early diagnostics?
- What has prevented you from suggesting testing to patients and/or their relatives? What has helped?
- What approach has been effective in encouraging patients to participate in the memory test?

**Ideas for improvements, perceived benefits and consequences of routine implementation­­:**

- Could you envision the long-term implementation of tablet-based testing in your practice? If yes/no, why/why not?
- What aspects would you improve, and why?
- How do you perceive the collaboration with the memory clinic? How are the referrals going?
- Have there been any patients who were not referred to the memory clinic despite an abnormal result? If so, why?
- What is needed for long-term and successful collaboration?
- What conditions are needed in your daily practice to provide better care for patients with memory impairments? What could be changed or improved?

**Conclusion of the interview:**

- Are there any aspects related to this topic that you think we haven't addressed yet?

**Interview guide: MAs (main questions)**

**Opening question:**

Since the winter of 2023, you and your practice have been participating in our study and have now had the opportunity to implement the testing with patients. Before you share your experiences with us, we would like to know whether and which tasks you have previously undertaken in the care of people with memory disorders, such as dementia.

**Role of the MA in dementia care:**

- How do you feel in the role of the testing MA? To what extent has your own understanding of your role or professional identity changed?
- How do patients respond to you as the administering MA?
- What impact do the tests have on your role as an MA? Have you noticed any changes regarding your colleagues (including supervisors), patients, and their relatives in relation to your role? If so, what changes have you observed?

**Experiences with the implementation of the dementia test via tablet:**

- How have you found the implementation of the tablet-based testing so far? What worked well, and what didn’t?
- How do you think the testing works with the tablet? For you, but also for the patients? What did you find easy? Are there any challenges or uncertainties?
- How was the test perceived by the patients?

**Benefits for patient care:**

- What impact has the project had on patient care?
- What benefits can you identify for your practice? What risks/negative aspects have you noticed?

**Ideas for improvements, perceived benefits and consequences of routine implementation**

- Could you imagine implementing tablet-based testing in your practice in the long term? If yes/no, why/why not?
- How has participation in the study influenced your personal attitude towards the dementia condition and dementia diagnostics?
- Occasionally, it is mentioned that dementia often remains undiagnosed in many patients for a long time. In your opinion, what would help to make early detection more effective and easier? What changes or improvments are needed?

**Conclusion of the interview:**

- Are there any aspects related to this topic that you think we haven't addressed yet?

**Interview guide: Patients (main questions)**

**Opening question**

- You recently participated in a memory test at your GP practice, which was administered by a medical assistant (MA) on a tablet. What prompted you to take part in the test?

**Personal experience of the test**

- How did you end up taking the test? Was it self-motivated, did someone approach you, or did family members suggest it?
- How did you feel beforehand? What ultimately persuaded you to take part in the test?
- How did you feel during the test? What did you find pleasant or interesting? Was there anything that bothered you? What surprised you during the appointment? Is there anything you wish you had known beforehand?
- How did you feel after the test? Did you feel well-informed about the next steps? If not, why not?
- Would you take the test again? Why or why not?

**User experience**

- How did you find the usability/operation of the tablet?
- What did you find easy? What challenges did you encounter? What made you feel uncertain?

**Testing by MA**

- How did you feel about your interaction with the MA who conducted the test? Was everything explained clearly, or was something unclear? What could be improved?
- It’s not yet common for MAs to carry out tasks like the test you took. What are your thoughts on this?

**Optional block of questions: Dementia and further care pathway for patients**

(These questions are only asked if patients bring up topics such as the clinical picture of dementia on their own initiative and/or ask specific questions to the interviewers)

- The test you participated in helps identify dementia and related changes at an early stage. What impact did the test and its results have on you?
- What steps were taken after the test? How did they progress? Was the further care pathway clear to you? Would you have liked more information at any point?
- How was the referral to the outpatient memory clinic? What went well, and what didn’t?

**Wishes, further comments**

- Your acquaintance is interested in taking a memory test but is unsure whether she should and has some concerns. What advice would you give her?
- Is there anything you would like to see in the care of people with memory disorders? What could be improved, either in general or in your GP practice?

**Conclusion of the inverview**

- What other aspects of the topic can you think of that we have not addressed so far?
